# Yoga for Healthy Aging: Science or Hype?

**DOI:** 10.20900/agmr20210016

**Published:** 2021-07-13

**Authors:** Purnima Madhivanan, Karl Krupp, Randall Waechter, Rahu Shidhaye

**Affiliations:** 1 Mel & Enid Zuckerman College of Public Health, University of Arizona, Tucson, Arizona 85724-5209, USA; 2 College of Medicine, University of Arizona, Tucson, Arizona 85724-5209, USA; 3 Public Health Research Institute of India, Mysore, Karnataka 560020, India; 4 School of Medicine, St. George’s University, Grenada, West Indies; 5 School of Graduate Studies, St. George’s University, Grenada, West Indies; 6 Pravara Institute of Medical Sciences, Loni 413713, India

**Keywords:** yoga, aging, mental health, physical health

## Abstract

Yoga, one of the world’s oldest health systems is receiving new attention for claims that it can contribute to healthy aging. Until recently, scientific evidence for its efficacy has relied heavily on small and poorly-designed research, but this is changing. Multiple, well-designed studies provide data showing that yoga practice has positive effects on cellular aging, mobility, balance, mental health, and prevention of cognitive decline—all areas of concern for older adults. Since the cost of implementing yoga-based community and home-based interventions is low—policymakers are also eyeing yoga practice as a cost-effective way to reduce medical costs and improve outcomes among a growing aging population. This commentary reviews the evidence for both physical and mental health benefits from yoga, as well as concerns about injuries that have been associated with certain types of yoga practice. It reveals a surprising range of yoga programs and difficulty levels that provide opportunities for almost anyone to participate and gain health benefits with practice.

Yoga’s roots can be traced to 1000 BCE and source texts with varying definitions for postures *(asanas)*, breath control, meditation, and spiritual practices. Most contemporary practice is based on Hatha yoga that is largely defined by physical practice [1]. The *Pradīpikā*, a Sanskrit manual written in 1350 describes fifteen primary postures, seven performed while seated, and eight in other positions, along with 69 combined postures for a total of 84 asanas [2]. Recent interventions have adapted these for a variety of health conditions problems in aging [3], pregnancy [4], chronic pain [5], diabetes [6], stroke [7], heart failure, mild traumatic brain injury and brain health in addition to many others [8,9]. More recent styles of yoga including *Vinyasa*, Iyengar, *Ashtanga, Kundalini*, and others have refined these postures, or put a varying emphasis on alignment, breath control, speed, and flow from one posture to another, adding various non-physical elements including meditation, relaxation, guided imagery, and commitments to particular diets and lifestyles [10]. Interventions using modified or adapted yoga postures and movements for people with medical limitations include Gentle Chair Yoga [11,12], group yoga interventions with psychoeducation for traumatic brain injury patients [13,14], *Hatha* yoga for increased balance and mobility in people 60 years and older [15], and Functional Fitness in adults with intellectual and developmental disabilities [16] among many others [17,18]. Intervention modifications depend on the medical condition; Prenatal Yoga is based on trimester and often eliminates positions like inversions to minimize risk of falling, and proning that may result in pressure point-related side effects [4]. Gentle Years Yoga for older adults uses a mixture of standing, seated, kneeling, supine, and prone stationary positions but alters *Hatha* Yoga poses to make them accessbile and safe for inactive older adults with comorbidities and physical and balance limitations [19]. Finally, some interventions focus on smaller movements, breathing and meditation that can safely be carried out either seated on a chair or in a wheel chair [11,12].

Many studies suggest that yoga practice is generally safe but as with any exercise, there are risks even for healthy people [20-22]. A survey of 2508 people with chronic diseases or ambulatory hospital outpatients attending yoga classes in Japan, and 271 yoga therapists, found that muscular pain was most common adverse even (5.3%), followed by joint pain (4.9%), muscle cramps (1.7%), dizziness (4.0%), numbness (1.9%), muscle twitching (1.6%), faintness (1.3%), heaviness of the head (1.0%), coughing (3.2%), nasal congestion (1.2%) runny nose (1.1%) [22]. The vast majority of adverse events were minor and transitory with only 1.9% of participants reporting adverse outcomes serious enough that they discontinued practice [22]. Studies suggest that almost all yoga postures can be modified and evidenced-based yoga programs can address specific deficits in strength and muscular endurance through the choice and modification of specific postures to reduce risk for adverse events and injuries [23]. Research also shows that that expert instruction and adaptation of poses to the limitations and needs of the participants with the use of blocks, straps, blankets, a support, or chair are useful and protective for users with physical limitations [24].

Recent research supports the hypothesis that yoga counteracts aging processes. Tolahunase and colleagues demonstrated that a 12-week intervention incorporating classical yoga postures, breathing exercises, and meditation was associated with positive changes in the levels of biomarkers of cellular aging including 8-OH2dG, a product of DNA damage; oxidative stress markers; and telomeres, the cellular clocks that shorten with each cell replication [25]. Santaella et al. examined the impact of long-term yoga practice on connectivity between the prefrontal and posterior cortex of the brain [26]—the network of interconnecting neurons that transfers data related to working-memory, spatial attention, and decision-making [26,27]. They showed that older women practicing yoga for at least 8 years had better functional brain connectivity compared with yoga-naive controls [28]. Cahn and colleagues found that a 90-day yoga and meditation retreat was associated with reductions in brain-derived neurotrophic factor, Hypothalamic-Pituitary Axis activity, increased IL-10 and decreased IL-12 indicators of lower overall inflammatory activity that has been associated with premature aging [29].

Yoga practice has also been shown to have positive neurological and mental health benefits [30,31]. A systematic review and meta-analysis of yoga practice on brain structure [31], found that regular practice was associated with anatomical changes in the frontal cortex, hippocampus, anterior cingulate cortex and insula—all areas implicated in aging-related cognitive decline. Other studies have similarly shown that yoga practice also has benefits for mental health. Gupta et al. found that yoga had both immediate and long-term impacts on State and Trait anxiety score [32]. Gururaja and colleagues showed that seniors age 65 to 75 years who participated in 90 minutes of yoga classes once or twice weekly for a month, had significant reductions in state and trait anxiety scores [33]. A systematic review and meta-analysis examining the impact of yoga practice on depression symptomatology also showed significant reductions (standardised mean difference = 0.41; 95% CI −0.65 to −0.17; *p* < 0.001) compared to waitlist controls, with increasing practice associated with additional benefits (β = −0.44, *p* < 0.01) [30]. Collectively, these findings suggest that yoga may be useful for mitigating age-related and neurodegenerative declines in older adults.

Yoga has also been found useful in maintaining physical mobility and functional independence in seniors [34]. A 10-country study of sedentary behavior among 350,000 adults age 60 and older, found that the average senior spends 9.4 h a day in sedentary activities—putting them at high-risk for premature aging [35,36]. Grabara and Szopa studied 56 women ranging in age from 50 to 79 years who attended a 20-week yoga retreat using pre- and post-measures of spine mobility [37] and found yoga practice was associated with greater muscle flexibility and a greater range of motion. Intervention participants demonstrated greater spinal mobility, more overall back strength, and more strength in their abdominal oblique muscles. In addition, a systematic review and meta-analysis of six clinical trials of yoga-based exercise interventions among individuals 60 and older, found an effect on balance (Hedges’ *g* = 0.40, 95% CI 0.15–0.65, 6 trials) and physical mobility (Hedges’ *g* = 0.50, 95% CI 0.06–0.95, 3 trials) [15]. Three trials in the review reported minor injuries including knee pain, lower back pain, and muscle strain; one reported a fall with no injuries, and two reported no adverse events. Beyond the physical benefits, yoga was also found to improve health related quality of life and mental well-being in older adults [38].

Finally, feasibility studies among older adults show high acceptability for yoga interventions [34,39]. Ranging in difficulty from ‘Chair yoga’ for older adults with mobility-impairments, to moderately strenuous traditional *Hatha* yoga for those that are more fit, almost all participants report feeling more limber and mobile, and many note reduction in chronic pain [39]. A study that examined willingness of seniors to engage in a yoga intervention also found that the top three barriers expressed were fears about the level of difficulty, a lack of motivation to engage in new practice, and fear of injury [40].

While everyone should consult their physician before starting a physical regimen, yoga appears to have a wide range of benefits including increased mobility; reduced risk for slip and fall; protection against cognitive decline; increased flexibility, strength, and balance; and improved sleep and mental well-being. The typical intervention is of moderate duration, around 45 min per week for 8 to 12 weeks. The range of intervention types and difficulty levels provide the opportunity for almost anyone to participate and gain health benefits. The quality of scientific evidence for traditional yoga practice is also improving with larger, better designed studies showing small to moderate benefits for most people in line with other forms of exercise programs [15]. Choosing an appropriate program for healthy aging come with the same cautions for any exercise in older adults [41]. Seniors are advised to start with lower-intensity activity and slowly increase the duration and difficulty to minimize risk of injury. Yoga practice offers both mental and physical benefits and different forms offer variations in emphasis [41]. For those more interested in cognitive benefits, ‘gentle yoga’ offers a slower pace and more focus on meditation and relaxation, while others may be more interested in improved flexibility or fitness, goals that would better be served by a more strenuous *Hatha* yoga program [42,43].
